# Cavernous Hemangioma of the Rib: A Rare Diagnosis

**DOI:** 10.1155/2010/254098

**Published:** 2010-05-31

**Authors:** Stavros Gourgiotis, Anastasios Piyis, Nikolaos Panagiotopoulos, Panayotis Panayotopoulos, Nikolaos S. Salemis

**Affiliations:** ^1^Second Surgical Department, 401 General Army Hospital of Athens, 41 Zakinthinou Street, Papagou, 15669 Athens, Greece; ^2^Thoracic Surgery Department, Euroclinic of Athens, 9 Athanasiadou Street, 11521 Athens, Greece

## Abstract

Hemangioma of the rib is an uncommon benign vascular tumour. A case of rib hemangioma in a 29-year-old woman is presented. Chest roentgenogram and computed tomography revealed a mass along the inner surface of the 7th left rib with bone destruction. She underwent resection of the 7th rib. The pathologic diagnosis was cavernous hemangioma. Hemangiomas of the rib are rare tumours but should be kept in mind in the differential diagnosis of rib tumours.

## 1. Introduction

Hemangioma of the rib is an uncommon benign vascular tumour with few cases reported in the literature [1–4]. It is reported that rib tumors represent 5.9% of the primary bone tumors and 89% of them are malignant [5]. Cavernous hemangioma of the rib is extremely rare and it should be considered in the differential diagnosis of rib tumours, especially in asymptomatic patients [2].

We, herein, describe an extremely rare case of a cavernous hemangioma of the rib which was found accidentally in a young female patient, the preoperative investigations, and the surgical treatment.

## 2. Case Presentation

**Figure 1:**
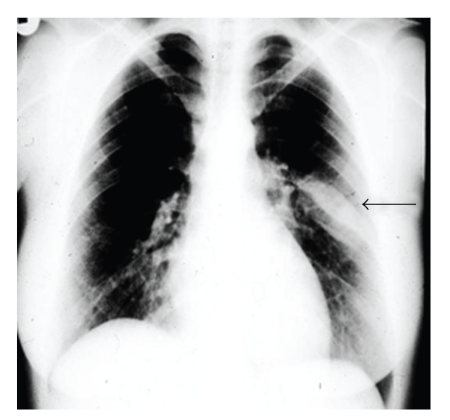
Chest X-ray shows a lesion in the 7th left rib.

**Figure 2:**
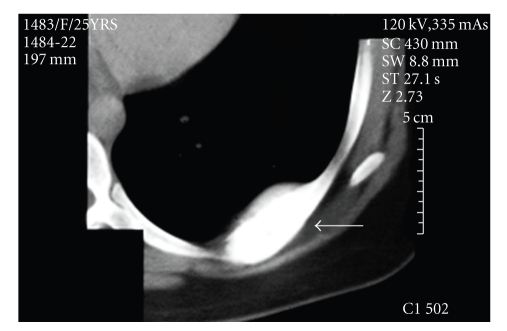
Computed tomography demonstrates a mass in the rib.

An asymptomatic 29-year-old female with no medical history or history of trauma to the chest wall was admitted due to a left chest wall mass which was incidentally discovered on routine chest X-ray (Figure 1). Computed tomography (CT) demonstrated a localized mass, measuring 4.5 cm, along the inner surface of the 7th left rib (Figure 2). Osteosclerosis was present on the top of the lesion along with calcification in different places and thickening on the nearby parietal pleura. Routine hematologic and blood biochemistry results were normal. The patient underwent left lateral minithoracotomy in which a total excision of the rib was performed. The patient had an uneventful recovery and discharged on the third postoperative day. Histological examination of the resected lesion revealed a cavernous hemangioma of the rib while the margins were negative for tumour cells.

## 3. Discussion

Bone hemangiomas represent about 1% of bone tumours [2]. Almost all these tumours occur in spine or in the skull; they are extremely rare in the ribs. Histologically, there are two types of hemangiomas: cavernous and capillary. The cavernous hemangiomas consist of large dilated vessels lined by a single layer of endothelial cells surrounded by a fibrous stroma layer while the capillary hemangiomas which are the less common, show numerous tortuous small vascular channels lined with epithelium. 

Patients are usually asymptomatic and the tumour is found incidentally on routine chest roentgenogram. Chest CT and magnetic resonance imaging can more clearly identify the size and the extent of cortical destruction. The differential diagnosis may include malignant rib tumours such as Ewing sarcoma, chondrosarcoma, and myeloma or benign rib tumours like ostheochondroma, fibrous dysplasia, eosinophilic granuloma, and aneurysmal bone cyst. 

Surgical excision of the rib is the treatment of choice while the histological examination reveals the diagnosis. Because more than half of primary rib tumours are malignant, prompt investigation, accurate tissue diagnosis, and surgical excision are required while preoperative biopsy in most cases of small chest tumours may be not helpful in diagnosis [2]. Furthermore, needle biopsy should be avoided due to the risk of bleeding or seeding the needle tract unless multiple myeloma or metastatic disease is highly suspected [1, 2, 5].
